# Etiologic Profile of Older Children With Diffuse Radiological Changes in Eastern China

**DOI:** 10.3389/fped.2022.823350

**Published:** 2022-05-02

**Authors:** Xuefeng Xu, Lingke Liu, Xuchen Xu, Qian Ma, Liping Teng, Haichun Zhou, Li Yang, Meiping Lu

**Affiliations:** ^1^Department of Rheumatology Immunology and Allergy, The Children's Hospital, Zhejiang University School of Medicine, National Clinical Research Center for Child Health, Hangzhou, China; ^2^Department of Radiology, The Children's Hospital, Zhejiang University School of Medicine, National Clinical Research Center for Child Health, Hangzhou, China

**Keywords:** children, diffuse radiological changes, etiology, high resolution computed tomography, interstitial lung disease

## Abstract

**Objective:**

To analyze the etiology of chest diffuse radiological changes (DRC) in children older than 2 years.

**Methods:**

A retrospective study was conducted on a primary cohort of children with DRC underwent high resolution computed tomography (HRCT).

**Results:**

DRC mainly included bronchial wall thickening, interlobular septal thickening, pleural thickening, ground glass opacity, mosaic perfusion, reticular & linear opacities, nodular opacity, and tree-in-bud. Of the identified 457 children with DRC, 83 of children older than 2 years with DRC were included in the present study. Ground glass opacity (53, 63.9%) and reticular & linear opacities (44, 53.0%) were frequently identified findings of HRCT, and no tree-in-bud pattern was observed. By contrast, among children with DRC by *M. pneumoniae* (*n* = 64), bronchial wall thickening (33, 51.6%), and mosaic perfusion (17, 26.6%) were common patterns of HRCT in addition to ground glass opacity (36, 56.3%). Most of etiologies were connective tissue disease (24, 28.9%), followed by diffuse alveolar hemorrhage syndrome (9, 10.8%), Langerhans cell histiocytosis (7, 8.4%), and recurrent aspiration (6, 7.2%).

**Conclusions:**

This study adds further insights into the role of HRCT in diagnosing childhood interstitial lung diseases, indirectly reflecting disease compositions.

## Introduction

Interstitial lung disease (ILD) in children (ChILD) represents a heterogeneous group of respiratory disorders that are mostly chronic and associated with high morbidity and mortality, encompassing more than 200 entities (1). ChILD is rare, and the estimated prevalence is <1 per 100 000 (2). Typical features of ILD include the presence of diffuse infiltrates on chest radiograph, and abnormal pulmonary function tests with evidence of a restrictive ventilatory defect and/or impaired gas exchange. For children, pulmonary function testing could be a major challenge, especially younger than 5 years. Furthermore, lung biopsy is also difficult to be accepted by their parents.

Advances in imaging diagnostic techniques have provided a better opportunity to study pulmonary diseases, especially the advent of high-resolution computed tomography (HRCT). The chest HRCT is the central diagnostic tool for ILD, contributing to discerning idiopathic pulmonary fibrosis from other forms of ILD (3). Importantly, chest HRCT could show a potential value in diagnosing ChILD, usually presenting with diffuse radiological changes (DRC). Clinical features and disease compositions of ChILD were previously described (1, 2, 4–6). However, reports on pediatric cases focused on imaging findings have been infrequently described. Moreover, the HRCT features associated with ChILD have not yet been assessed. So far, data on etiology of chest DRC in children, especially in children older than 2 years, is extremely limited. The present study aimed to describe the etiologic profile of children with DRC, revealing possible disease spectrum composition of ChILD in eastern China. Given the high incidence of *Mycoplasma pneumoniae* (*M. pneumoniae*) pneumonia in Chinese children, we also analyzed the characteristics of DRC caused by *M. pneumoniae* and made a comparison between the two groups.

## Methods

### Study Population

**Figure 1:**
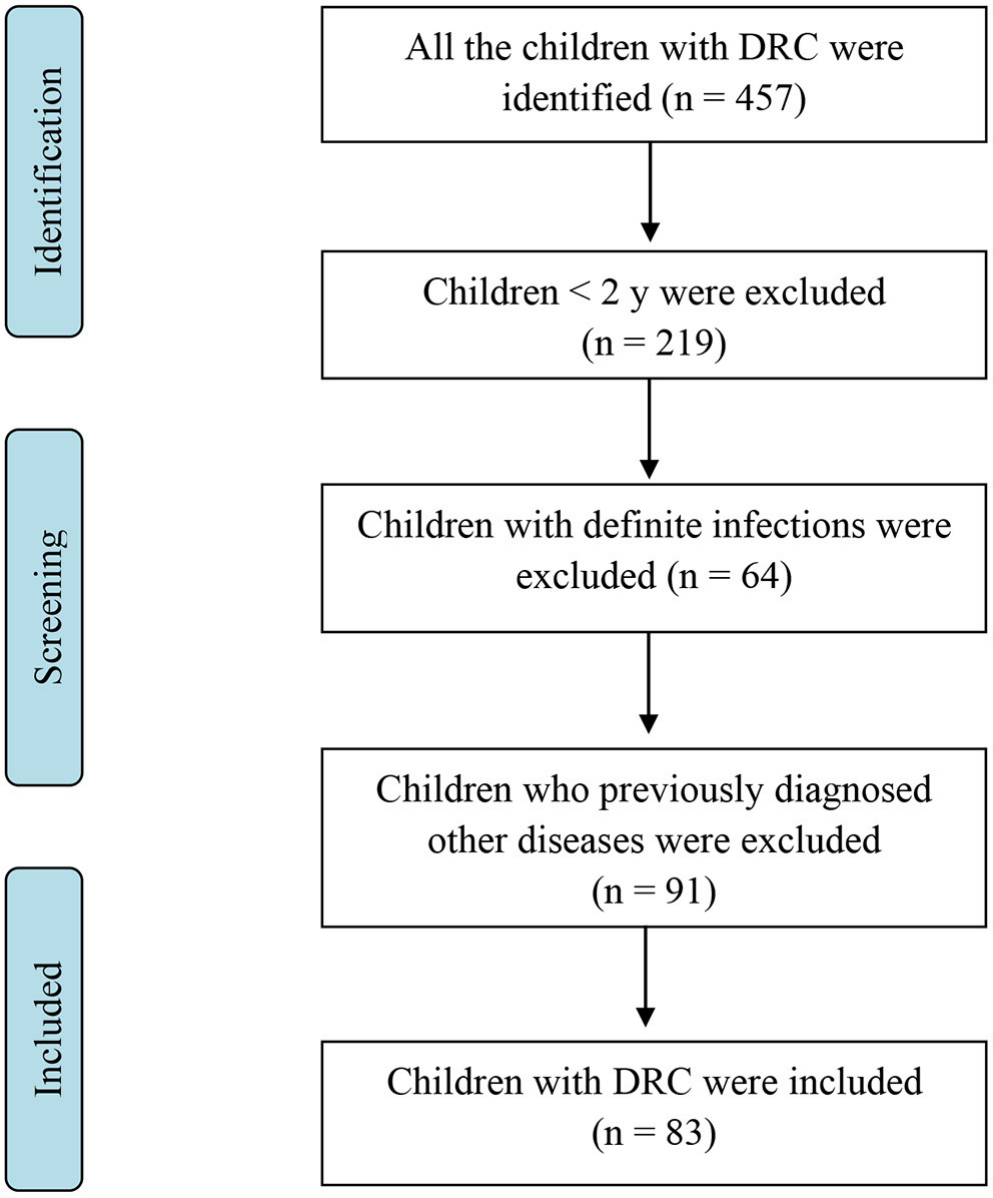
Patient selection flow chart.

This was a single-center retrospective study designed to assess the etiologic profile of children with DRC underwent HRCT. Study participants were selected from electronic medical records using a computer-assisted search function of National Clinical Research Center, the Children's hospital, Zhejiang University School of Medicine (Hangzhou, China), from January 2015 to December 2019. 457 children with DRC were identified from the HRCT reports (Figure 1). The exclusion criteria were: (1) those DRC patients with definite infections including tuberculosis or HIV; (2) those with chronic lung diseases, preterm, congenital heart disease; (3) those previously diagnosed tumors; (4) those with DRC caused by a clear disease; and (5) children with younger than 2 years. To better understand the features of DRC, we also analyzed the characteristics of DRC caused by *Mycoplasma pneumoniae*. This study was approved by the Ethic Review Board of Children's Hospital, Zhejiang University School of Medicine.

### Radiological Assessment

Two pediatric radiologists independently examined the chest HRCT during hospitalization and analyzed all images and reached consensus conclusions. Patterns of DRC mainly included bronchial wall thickening, interlobular septal thickening, pleural thickening, ground glass opacity, mosaic perfusion, reticular & linear opacities, nodular opacity, and tree-in-bud (7, 8).

### Statistical Analysis

All the statistical analysis and graphics were performed with R statistical software packages (R version 3.4.3). Descriptive statistics (frequency, median, and interquartile range) were calculated. The continuous variables between groups were compared by Student's *t*-test or Wilcoxon Rank test. For categorical variables, Pearson's chi-squared test was applied. A *P*-value of < 0.05 was considered statistically significant in all the analysis.

## Results

### Clinical Features

Figure 1 shows the patient's selection process. Of the identified 457 children with DRC, 83 of children older than 2 years with DRC were included in the present study (Table 1). The median duration of symptoms was 2 months. Cough and/or wheeze were the most common manifestations, followed by tachypnea, fever, fatigue, and joint pain. We also analyzed patients with DRC caused by *Mycoplasma pneumoniae* (*M. pneumoniae*) infections, showing a different clinical feature (Table 1). All the Children with DRC by *M. pneumoniae* presented with cough or wheeze, followed by fever, and tachypnea.

**Table 1:** Clinical characteristic of 83 children with diffuse radiological changes (DRC).

	**Children**	***M. pneumoniae*** **-**
**Characteristics**	**with DRC**	**associated DRC**	***P-*** **value**
No. of patients	83	64	-
Age, (median, year)	4.05 (2–15)	4.32 (2–12.8)	-
Gender			0.867
Boy	37 (44.6%)	27 (42.2%)	
Girl	46 (55.4%)	37 (57.8%)	
Delivery mode			0.134
Vaginal delivery	42 (50.6%)	25 (39.1%)	
Cesarean section	41 (49.4%)	39 (60.9%)	
Duration of symptoms (median)	2m (2 d−60 m)	8d (2 d−29 d)	
Symptoms (frequency)			
Cough/wheeze	53 (63.8%)	64 (100%)	<0.001^**^
Tachypnea	27 (32.5%)	16 (25.0%)	0.364
Fatigue	18 (21.7%)	3 (4.7%)	0.004^**^
Fever	16 (19.3%)	52 (81.3%)	<0.001^**^
Joint pain	11 (13.3%)	3 (4.7%)	0.095
Rash	9 (10.8%)	14 (21.9%)	0.108
Hemoptysis/	8 (9.6%)	3 (4.7%)	0.349
Hematemesis			
Short of breath	6 (7.2%)	2 (3.1%)	0.466
Pale	6 (7.2%)	1 (1.6%)	0.138

** *P < 0.01*.

### Patterns of Radiological Imaging

**Figure 2:**
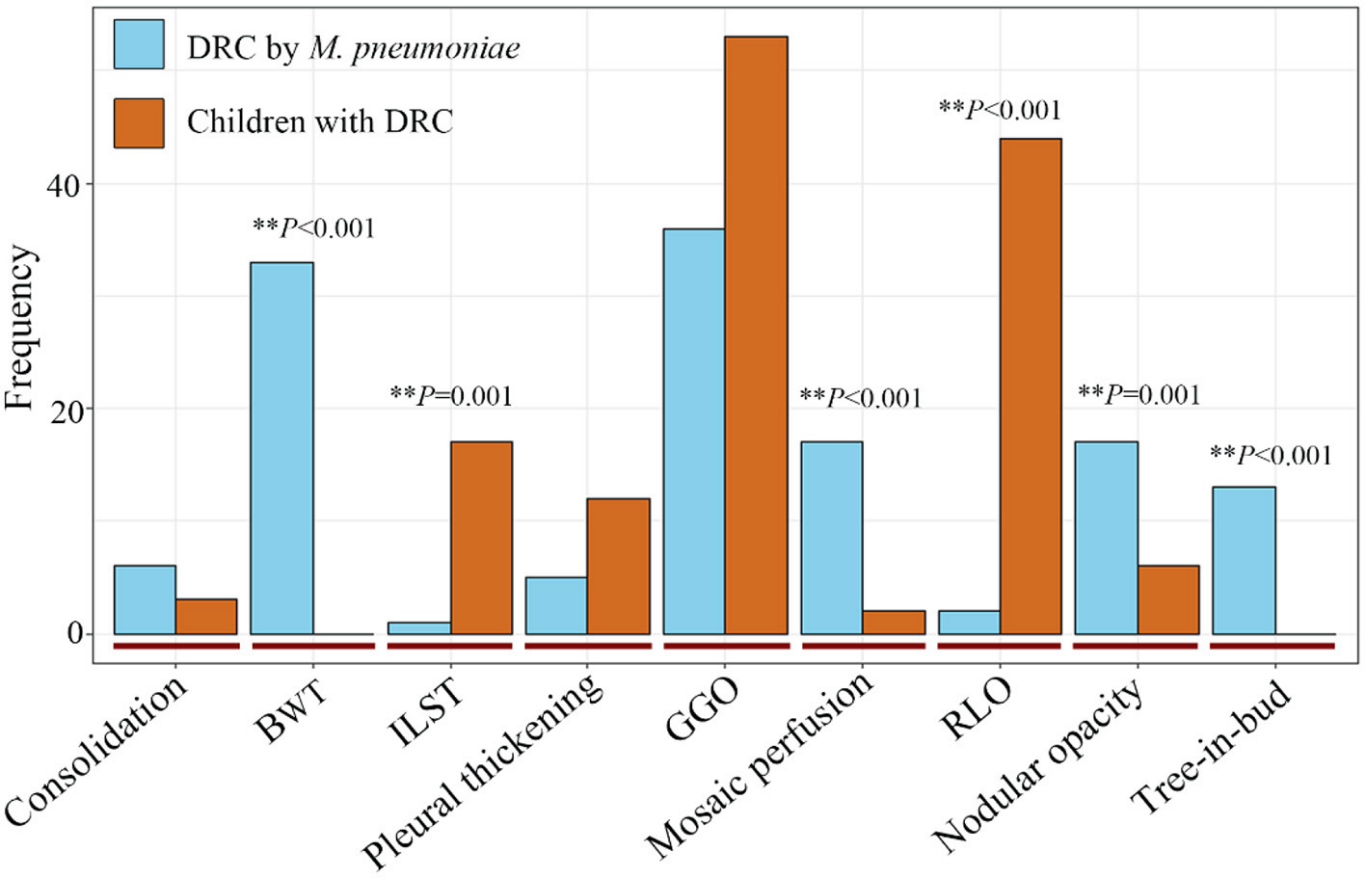
HRCT features of diffuse radiological changes (DRC) in children older than 2 years. Among children with DRC, ground glass opacity and reticular & linear opacities were most frequently identified imaging findings, followed by interlobular septal thickening and pleural thickening. They had no tree-in-bud pattern and bronchial wall thickening. Notably, children with DRC caused by *M. pneumoniae* infections showed a different pattern. In addition to ground glass opacity, they presented with bronchial wall thickening, mosaic perfusion, and tree-in-bud. BWT, bronchial wall thickening; ILST, interlobular septal thickening; GGO, ground glass opacity; RLO, reticular and linear opacities. **P < 0.01.

**Figure 3:**
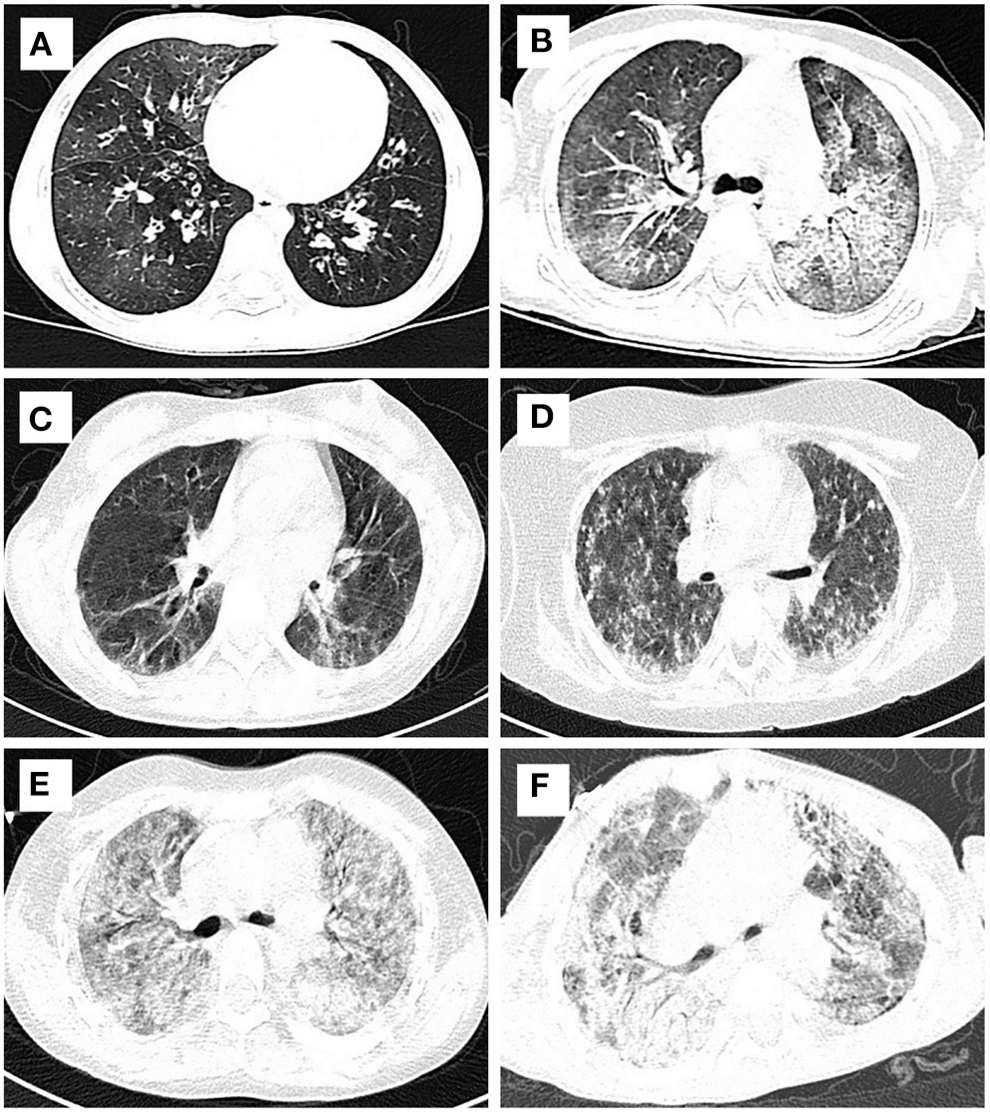
Typical HRCT imaging of patients with DRC. Mosaic perfusion, interlobular septal thickening and ground glass opacity from bronchiolitis obliterans **(A)** and alveolar proteinosis **(B)**; reticular and linear opacities and pleural thickening from juvenile dermatomyositis **(C)** and pulmonaty fibrosis **(D)**; ground glass opacity, interlobular septal thickening, and reticular & linear opacities from diffuse alveolar hemorrhage syndromes **(E)** and Sting associated with vasculopathy with onset in infancy **(F)**.

Ground glass opacity (63.9%), reticular & linear opacities (53.0%), and interlobular septal thickening (20.5%) were frequently identified findings of HRCT among children with DRC, and no tree-in-bud pattern was observed (Figures 2, 3). However, among children with DRC by *M. pneumoniae*, imaging patterns of HRCT demonstrated a significant difference. In addition to ground glass opacity (56.3%), bronchial wall thickening (51.6%) and mosaic perfusion (26.6%) were common patterns of HRCT. Tree-in-bud (20.3%) was also observed, indicating an infection specific pattern. Interlobular septal thickening (20.5 vs. 1.6%, *P* = 0.001) and reticular & linear opacities (53.0 vs. 3.1%, *P* < 0.001) were more common in children with DRC, while bronchial wall thickening (61.6 vs. 0%, *P* < 0.001), and tree-in-bud (20.3 vs. 0%, *P* < 0.001) were frequently seen among DRC children by *M. pneumoniae* (Figure 2).

### Disease Compositions of Children With DRC

Systemic disease-associated ILD were the most common causes, accounting for 67.5% of the patients, followed by alveolar structure disorder-associated ILD (18.1%), and exposure related ILD (13.3%). Among systemic disease associated ILD, most of etiologies was connective tissue disease including juvenile dermatomyositis, systemic lupus erythematosus, and juvenile idiopathic arthritis, followed by Langerhans cell histiocytosis, and leukemia (Table 2).

**Table 2:** Etiology of children with diffuse radiological changes.

**Identified orders**	**No. (%)**
Systemic disease-associated	56 (67.5)
Juvenile dermatomyositis	5
Systemic lupus erythematosus	7
Juvenile idiopathic arthritis	6
ANCA-associated vasculitis	4
Interstitial pneumonia with autoimmune features	2
Nephritis/nephrotic syndrome	4
Langerhans cell histiocytosis	7
Leukemia	5
Lymphoma	3
Primary immunodeficiency	3
High IgM/IgE syndrome	3
Niemann-Pick disease	3
Other	4
Exposure-related	11 (13.3)
Hypersensitivity pneumonitis	3
Recurrent aspiration	6
Drug-induced	2
Alveolar structure disorder-associated	15 (18.1)
Alveolar proteinosis	3
Diffuse alveolar hemorrhage syndromes	9
Other	3
Unclassified	1 (1.1)

## Discussion

The present study explored the etiologic composition of ChILD from the perspective of HRCT. The most frequently identified patterns of HRCT in children with DRC were ground glass opacity and reticular & linear opacities without tree-in-bud pattern, and most of etiologies were connective tissue disease.

For children with DRC, imaging patterns of HRCT will be more likely to show subacute and chronic lesions or fibrosis process, significantly different from those induced by *M. pneumoniae*. Our patients with DRC did not include those children who had previously diagnosed tumors, preterm infants, or other definite secondary DRC, possibly leading to a potential bias. In clinical practices, the development of DRC caused by preterm or gene mutation occurred mainly in neonates and infants (<2 years) (4). Therefore, the present study mainly focused on the etiologic spectrum of ChILD in children older than 2 years old. On the other hand, *M. pneumoniae* infection was the main cause of community-acquired pneumonia in China (9); interstitial pneumonia by *M. pneumoniae* was frequently found (10). To better understand the features of DRC, we also analyzed the imaging features of interstitial pneumonia by *M. pneumoniae*. We found that ground grass opacity was most frequently seen in children with DRC and that by *M. pneumoniae*. *M. pneumoniae* specific bronchial wall thickness and tree-in-bud were less frequently observed in children with DRC, indicating a significant difference between the two groups. Additionally, children with DRC were more likely to manifest as fatigue, suggesting an insidious progression. This would make the early diagnosis of ChILD more difficult.

There is increasing evidence that the etiologies of ILD in infants under 2 years are significantly distinct from those in older children and adults (4). Diffuse developmental disorders, growth abnormalities, and gene mutations are more common causes of ChILD in neonates and infants (4, 6, 11). However, some definite entities associated with ChILD, including cystic fibrosis, primary immunodeficiency, congenital heart disease, bronchopulmonary dysplasia, pulmonary infection, and primary ciliary dyskinesia were not taken into consideration. For children older than 2 years of age, different studies described different etiologic profile. Rice et al. presented 118 cases underwent lung biopsies. They found that the most common diseases were airway diseases such as follicular bronchiolitis and chronic bronchiolitis, followed by non-specific interstitial pneumonia, haemosiderosis, and hypersensitivity pneumonitis; which overlapped with those seen in adults (12). Another multicenter interdisciplinary study included 191 biopsy-proven ChILD in North America, demonstrating that infectious or postinfectious etiology was most common diagnosis, followed by disorders related to systemic disease among the immunocompetent host (11). While opportunistic infections and disorders related to treatment (including chemotherapy, radiation, and combined therapy) and drug were common diagnostic category among the immunocompromised children (11).

Our study analyzes the composition of etiology from the perspective of HRCT, regardless of with or without biopsy. This study showed that systemic disease associated ILD were the most common causes, followed by alveolar structure disorder associated ILD and exposure related ILD. Furthermore, among systemic disease associated ILD, most of etiologies was connective tissue disease. This result was similar to the previous report by Tang et al. (5) presenting with the common causes of systemic disease associated ILD, alveolar structure disorder associated ILD and exposure related ILD. These studies also indicated that HRCT may also reflect the presence of ILD and disease composition of ChILD. The present study differed from those studies mentioned above, predominant in airway diseases and infectious diseases. This phenomenon could be associated with different inclusion criteria. For example, we ruled out children with postinfectious DRC like bronchiolitis obliterans caused by severe adenovirus infections.

Notably, mutations in the gene encoding surfactant protein have been frequently identified in neonate and infants. Some children were sporadic cases with de novo mutations, and the other had inherited the mutation from a parent (13, 14). Our study showed that alveolar proteinosis can occur not only in neonates or infants, but also in children older than 2 years. Furthermore, a study by Moorsel et al. (15) showed that surfactant protein C gene mutations are responsible for disease development in about a quarter of adult cases with familial pulmonary fibrosis in a Dutch cohort, presenting with non-classifiable HRCT chest scan patterns with cystic changes. This further suggested that disorders more prevalent in infancy such as diffuse developmental disorders, growth abnormalities, and surfactant dysfunction mutations, may also occur in older children and adults, which making the etiological diagnosis of ILD more challenging.

The present study has several limitations. First, its single-center and retrospective design could limit external applicability. However, our children's hospital belongs to a general and tertiary hospital, and our patients come from Zhejiang Province and its surrounding areas, like that in a population-based study. Second, for adults, HRCT scanning was easily performed, whether it is inspiratory or expiratory. Children cannot hold their breath during HRCT examination, especially younger children, which will lead to a selection bias. Nevertheless, combined with the patient's symptoms, signs, and other examination results, these patients can also be accurately diagnosed.

In conclusion, we reported imaging features and etiologic compositions of children with DRC in East China. Our study adds further insights into the role of HRCT in diagnosing ChILD, indirectly reflecting disease compositions of ChILD.

## Data Availability Statement

The original contributions presented in the study are included in the article/supplementary material, further inquiries can be directed to the corresponding author.

## Ethics Statement

The studies involving human participants were reviewed and approved by the Ethic Review Board of Children's Hospital, Zhejiang University School of Medicine.

## Author Contributions

XueX, LL, and ML designed the study, interpreted the data and critically reviewed, and revised the manuscript. XucX, QM, and LT undertook data collection and critically reviewed, and revised the manuscript. LY and HZ analyzed the chest imaging, contributed to analyses and data interpretation, and critically reviewed and revised the manuscript. All authors contributed to the article and approved the submitted version.

## Funding

This work was supported by fund from the National Natural Science Foundation of China (No. 81871220).

## Conflict of Interest

The authors declare that the research was conducted in the absence of any commercial or financial relationships that could be construed as a potential conflict of interest.

## Publisher's Note

All claims expressed in this article are solely those of the authors and do not necessarily represent those of their affiliated organizations, or those of the publisher, the editors and the reviewers. Any product that may be evaluated in this article, or claim that may be made by its manufacturer, is not guaranteed or endorsed by the publisher.
